# Stent Patency and Survival after PTBD and Biliary Stenting for Pancreatic Cancer: A 5-Year Retrospective Cohort Study

**DOI:** 10.34172/aim.34988

**Published:** 2025-12-01

**Authors:** Yun Tao, Jie Tang, Wenhui Yu, Wenge Yang, Meng Zhang, Qinghua Wu, Jie Li

**Affiliations:** ^1^Department of Interventional Radiology, Affiliated Hospital of Jiangnan University, 214000, Wuxi, China

**Keywords:** Biliary stent, Interventional radiology, Obstructive jaundice, Palliative care, Pancreatic cancer, PTBD, Stent patency, Survival analysis

## Abstract

**Background:**

Obstructive jaundice commonly complicates pancreatic cancer and often requires biliary decompression. Percutaneous transhepatic biliary drainage (PTBD) followed by stent placement is used for palliation, but long-term stent patency and the relationship between patency and overall survival (OS) remain incompletely characterized.

**Methods:**

We conducted a retrospective cohort study of 60 consecutive patients who underwent sequential PTBD and biliary stent placement at the Affiliated Hospital of Jiangnan University (Wuxi, China) between January 2020 and December 2024. Primary endpoint was stent patency (time from stent insertion to radiologically confirmed occlusion or repeat intervention). Secondary endpoint was OS measured from stent insertion. Patient characteristics, stent type (covered vs uncovered), tumor location, stage, and receipt of systemic chemotherapy were extracted from electronic medical records. Kaplan–Meier analysis and Cox proportional hazards models (adjusted for age, sex, cancer stage, tumor location, baseline bilirubin and chemotherapy) were used. Proportional hazards assumption was tested using Schoenfeld residuals.

**Results:**

Median stent patency was 12.0 months (IQR 8.0–15.0) and median OS was 9.5 months (IQR 6.0–13.0). Covered stents were associated with longer patency (median 13.0 vs 11.0 months; log-rank *P*=0.018). In multivariable Cox regression, Stage IV disease (adjusted HR 2.50; 95% CI 1.68–3.86; *P*<0.001) and age (per year, adjusted HR 1.05; 95% CI 1.02–1.09; *P*=0.002) were independent predictors of mortality; covered stent use was associated with lower mortality (adjusted HR 0.78; 95% CI 0.61–0.99; *P*=0.043). Schoenfeld tests showed no violation of the proportional hazards assumption (global *P*=0.18). Stent-related complications occurred in 16.7% of patients (migration 5.0%, infection 3.3%, biliary leak 1.7%, recurrent jaundice 6.7%).

**Conclusion:**

Sequential PTBD and biliary stenting provides effective biliary decompression with a median stent patency of 12 months but only limited impact on OS, which is dominated by disease stage. Covered stents improved patency and were associated with a modest survival advantage after adjustment. Prospective, multicenter studies are required to confirm these findings and to explore integration with systemic therapies.

## Introduction

 Pancreatic cancer is a leading cause of cancer mortality worldwide; recent estimates place it among the top four causes of cancer-related deaths in many regions, with a 5-year survival below 10% in most series.^1-3^ Obstructive jaundice is a frequent manifestation, particularly for tumors of the pancreatic head, and contributes substantially to morbidity and delays in systemic therapy.^4^ While endoscopic retrograde cholangiopancreatography (ERCP) with stent placement is first-line in many centers, PTBD remains an important alternative where ERCP is not feasible or has failed.^2,5^ According to the World Health Organization, it ranks fourth in global cancer-related mortality; in East Asia, the incidence continues to rise, reaching over 8 cases per 100,000 population annually.^6^ A significant proportion of patients present with obstructive jaundice, a condition resulting from bile duct compression by the tumor, particularly when the neoplasm is located at the head of the pancreas. This leads to impaired bile drainage, causing clinical symptoms such as jaundice, pruritus, fatigue, weight loss, and metabolic disturbances.^7,8^ Management of malignant obstructive jaundice is a critical component of palliative care in pancreatic cancer, aiming to alleviate the symptoms and improve quality of life.^9^ One widely employed approach is percutaneous transhepatic biliary drainage (PTBD), often followed by the placement of biliary stents to maintain bile flow.^10^ Several studies have highlighted the effectiveness of PTBD and stenting in reducing bilirubin levels, relieving jaundice-related symptoms, and improving nutritional status.^11,12^

 While short-term outcomes of PTBD-stent placement have been well-documented, including symptom relief and reduced complication burden compared to surgical alternatives, its long-term impact on stent patency and survival remains unclear. Stent occlusion due to tumor ingrowth, migration, or sludge formation remains a frequent complication, often necessitating re-intervention.^13^ Factors such as tumor size, location, and the patient’s performance status also influence stent durability and clinical outcomes.^14^ Evidence from recent literature presents mixed results. Some studies have reported median stent patency of 8–12 months, while others have suggested extended patency of up to 16 months with covered metal stents.^14,15^ Covered stents, in particular, show promise in minimizing tumor ingrowth and prolonging lumen patency. Nevertheless, the extent to which stent patency correlates with overall survival (OS) remains a subject of debate.^16,17^ Although stenting improves symptom control, it does not appear to significantly prolong life expectancy in many patients. Moreover, the aggressive nature of pancreatic cancer, especially in advanced stages, often limits the survival benefits of biliary drainage alone. As such, integrating PTBD-stenting with adjunct treatments (such as chemotherapy, radiotherapy, or emerging targeted therapies) has become an area of growing interest.^18^ Nonetheless, the literature lacks sufficient high-quality, long-term data specifically evaluating survival and stent outcomes beyond one year in this patient population.^19^ Understanding the long-term outcomes of different stent types may help optimize palliative strategies, particularly in settings where resource limitations and access to endoscopic interventions restrict treatment choices. Evidence on whether covered stents justify higher costs by improving patency and survival can guide stent selection and healthcare planning.

 To address this gap, the present study aims to evaluate the long-term patency and survival outcomes of PTBD-stent treatment in patients with pancreatic cancer-related obstructive jaundice, over a five-year follow-up period. By analyzing real-world clinical data, this study seeks to contribute evidence that may guide future palliative strategies and improve decision-making in managing this challenging condition.^20^ However, long-term data on stent patency after PTBD and the relationship between patency and OS is limited, especially from single-center cohorts in East Asia. The primary objective of this study was to evaluate long-term biliary stent patency after sequential PTBD–stent therapy. Secondary objectives included assessment of OS, complication rates, and predictors of stent failure and mortality. We hypothesized that covered self-expanding metal stents would provide longer patency than uncovered stents, but survival would remain primarily determined by disease stage.

## Materials and Methods

###  Study Design and Population

 This retrospective cohort study was designed and reported in accordance with the STROBE (Strengthening the Reporting of Observational Studies in Epidemiology) statement and the RECORD (REporting of studies Conducted using Observational Routinely-collected Data) extension. This study employed a retrospective cohort design to investigate the long-term outcomes of PTBD followed by stent placement in patients with pancreatic cancer-related obstructive jaundice. This is a retrospective cohort study including all consecutive patients who underwent sequential PTBD followed by biliary stent placement for pancreatic cancer–associated obstructive jaundice at the Affiliated Hospital of Jiangnan University between January 1, 2020 and December 31, 2024. In total, 60 patients who underwent PTBD followed by biliary stent placement were identified and included. A total of 76 patients with pancreatic cancer–associated biliary obstruction were screened during the study period. Sixteen were excluded (five due to non-pancreatic malignancy, four for lack of follow-up data, and seven for PTBD without stent placement), leaving 60 eligible patients for final analysis. Among the included patients, a small subset with stage I–II disease did not undergo curative surgery. These patients were considered medically unfit due to comorbidities or advanced age, or they declined surgical resection after multidisciplinary evaluation. In a few cases, initial imaging suggested resectability, but subsequent intraoperative or radiological findings demonstrated vascular encasement or local extension precluding surgery; thus, they were managed palliatively with PTBD and stent placement.

###  Inclusion and Exclusion Criteria 

 Inclusion criteria: Age ≥ 18 years; clinical and/or histopathological diagnosis of pancreatic ductal adenocarcinoma; radiological or clinical evidence of obstructive jaundice; and treatment with PTBD followed by biliary stent insertion during the study period.

 Exclusion criteria: Biliary obstruction due to non-pancreatic malignancy; patients receiving only PTBD without stent placement; follow-up < 6 months post-intervention unless death occurred within 6 months; and insufficient clinical records for endpoint ascertainment. Institutional ethics committee approval was obtained prior to data collection. All patient data were anonymized in accordance with confidentiality and privacy regulations.

###  Case Identification and Data Source

 Patients were identified from the interventional radiology procedure logs and the hospital electronic medical record system (HIS). Clinical diagnoses were confirmed by review of imaging reports, pathology (when available), and treating clinicians’ documentation. Data extracted included demographics, tumor location and stage, baseline laboratory values (including bilirubin), details of PTBD and stent type (covered vs. uncovered), interval between PTBD and stent placement, complications, receipt of systemic chemotherapy, and survival status.

###  PTBD and Stent Placement Procedure

 All procedures were conducted by experienced interventional radiologists under fluoroscopic guidance. In our institution, PTBD is the standard first-line approach for malignant biliary obstruction, particularly in cases where ERCP is unavailable after hours, technically unfeasible, or contraindicated due to altered anatomy (e.g. post-gastrectomy). Stent placement was performed after initial drainage once bilirubin levels improved and the tract had matured. PTBD was performed under local anesthesia and conscious sedation. A biopsy needle was inserted percutaneously through the liver parenchyma to access the obstructed bile duct. Once the duct was cannulated, a guidewire was introduced to confirm placement, followed by catheter insertion for biliary drainage. Subsequently, endoscopic biliary stenting was performed using either covered or uncovered self-expanding metal stents, as deemed appropriate by the treating physician based on tumor location, morphology, and patient condition. Covered stents were preferentially used in cases of suspected tumor ingrowth. Primary outcome (stent patency) was defined as the time from initial stent insertion to documented stent occlusion, defined by (a) recurrence or worsening of cholestatic symptoms with a bilirubin rise to > 2 mg/dL or > 2 × baseline, and (b) radiologic evidence of stent obstruction (US/CT/MRCP) or need for repeat intervention (exchange or re-stenting). Secondary outcome (overall survival) was defined as time from stent insertion to death from any cause. Patients alive at the end of the study (December 31, 2024) or lost to follow-up were censored at last known contact. Patients without documented occlusion by the end of the observation period were censored at the date of last radiologic or clinical assessment.

###  Data Collection and Follow-up

 Patient demographic and clinical data, including age, sex, comorbidities, tumor location (head, body, or tail of the pancreas), disease stage, and presence of metastasis, were extracted from electronic medical records. Follow-up assessments were conducted at three-month intervals for the first two years and biannually thereafter. These evaluations included liver function tests (serum bilirubin, ALT, AST), tumor biomarkers, and imaging studies (ultrasound or CT scan) to assess stent patency and tumor progression. Stent patency was defined as the duration from the initial stent placement to either documented stent occlusion or recurrence of jaundice symptoms confirmed radiologically. Complications such as infection, bile leakage, or stent migration were also documented. The interval between PTBD and definitive stent placement was recorded. Stent type (covered vs. uncovered self-expanding metal stent) was chosen by the treating interventional radiologist based on tumor anatomy, perceived risk of tumor ingrowth, prior interventions, and device availability. This decision was generally physician-driven rather than randomized.

###  Statistical Analysis and Cox Model Details

 Descriptive statistics are presented as mean ± SD or median (IQR) for continuous variables and counts (percentages) for categorical variables. Kaplan–Meier curves and log-rank tests compared time-to-event outcomes. Multivariable Cox proportional hazards models assessed predictors of survival and patency; candidate covariates included age, sex, tumor stage (I–IV), tumor location (head vs body/tail), stent type (covered vs. uncovered), baseline bilirubin, and receipt of systemic chemotherapy (yes/no). Covariates were selected *a priori* based on clinical relevance. Proportional hazards assumptions were tested using Schoenfeld residuals and inspection of log-log survival plots. A two-sided *P* < 0.05 was considered statistically significant. Analyses were performed in IBM SPSS Statistics (version 22.0).

###  Ethical Considerations

 Given the retrospective design, informed consent was waived. All data were de-identified to preserve patient confidentiality. Patients were censored at the date of last clinical contact or at the end of study follow-up (December 31, 2024). Death dates were extracted from medical records and the hospital death registry when available. The study adhered to ethical standards outlined by the institutional review board and complied with international privacy regulations, including HIPAA and GDPR. The study protocol was approved by the Ethics Committee of the Affiliated Hospital of Jiangnan University (Approval No. LS2024556; Decision No. SL2024285; 17 December 2024). The need for informed consent was waived because of the retrospective design and de-identified data analysis.

## Results

###  Patient Demographics and Clinical Characteristics

 In the present study, 60 patients with pancreatic cancer and obstructive jaundice were included in the study, for whom PTBD and stent placement was performed. Among the included patients, the mean age was 64.5 ± 9.2 years with a slight male domination as 31 (51.7%) were men and 29 (48.3%) were women. The patients’ cancer stages were distributed as follows; 15 (25%) were diagnosed with stage one pancreatic cancer, 18 (30%) were diagnosed with stage two pancreatic cancer, 16 (26.7%) had stage three pancreatic cancer and 11 (18.3%) had stage four pancreatic cancer. Regarding the anatomic distribution of tumors within the pancreas, 22 patients had tumors in the head of the pancreas, 19 patients had tumors in the body, and 19 patients had tumors in the tail of pancreas (36.7%, 31.7%, and 31.7%, respectively). Among these, 30 patients received covered metallic stents and the other 30 patients received the uncovered ones. The patient characteristics are presented in Table 1.

**Table 1:** Demographic Features and Baseline Clinical Characteristics of the Study Population

**Variable**	**Total (n=60)**	**Covered stent (n=30)**	**Uncovered stent (n=30)**
Age, mean ± SD	64.5 ± 9.2	64.1 ± 8.7	65.0 ± 9.6
Age, median (IQR)	65 (58–72)	64 (57–70)	66 (59–73)
Male sex — n (%)	31 (51.7)	16 (53.3)	15 (50.0)
Stage I — n (%)	15 (25.0)	7 (23.3)	8 (26.7)
Stage II — n (%)	18 (30.0)	8 (26.7)	10 (33.3)
Stage III — n (%)	16 (26.7)	8 (26.7)	8 (26.7)
Stage IV — n (%)	11 (18.3)	7 (23.3)	4 (13.3)
Tumor location — Head/Body/Tail — n (%)	22/19/19 (36.7/31.7/31.7)	11/9/10 (36.7/30/33.3)	11/10/9 (36.7/33.3/30)
Baseline total bilirubin, median (IQR)	8.5 (6.2–12.1)	8.3 (6.0–11.8)	8.7 (6.4–12.5)
Received systemic chemo — n (%)	36 (60%)	18 (60%)	18 (60%)
Prior ERCP attempt — n (%)	12 (20%)	5 (16.7%)	7 (23.3%)

Note: A total of 60 patients with pancreatic cancer-associated obstructive jaundice were enrolled

**Figure 1:**
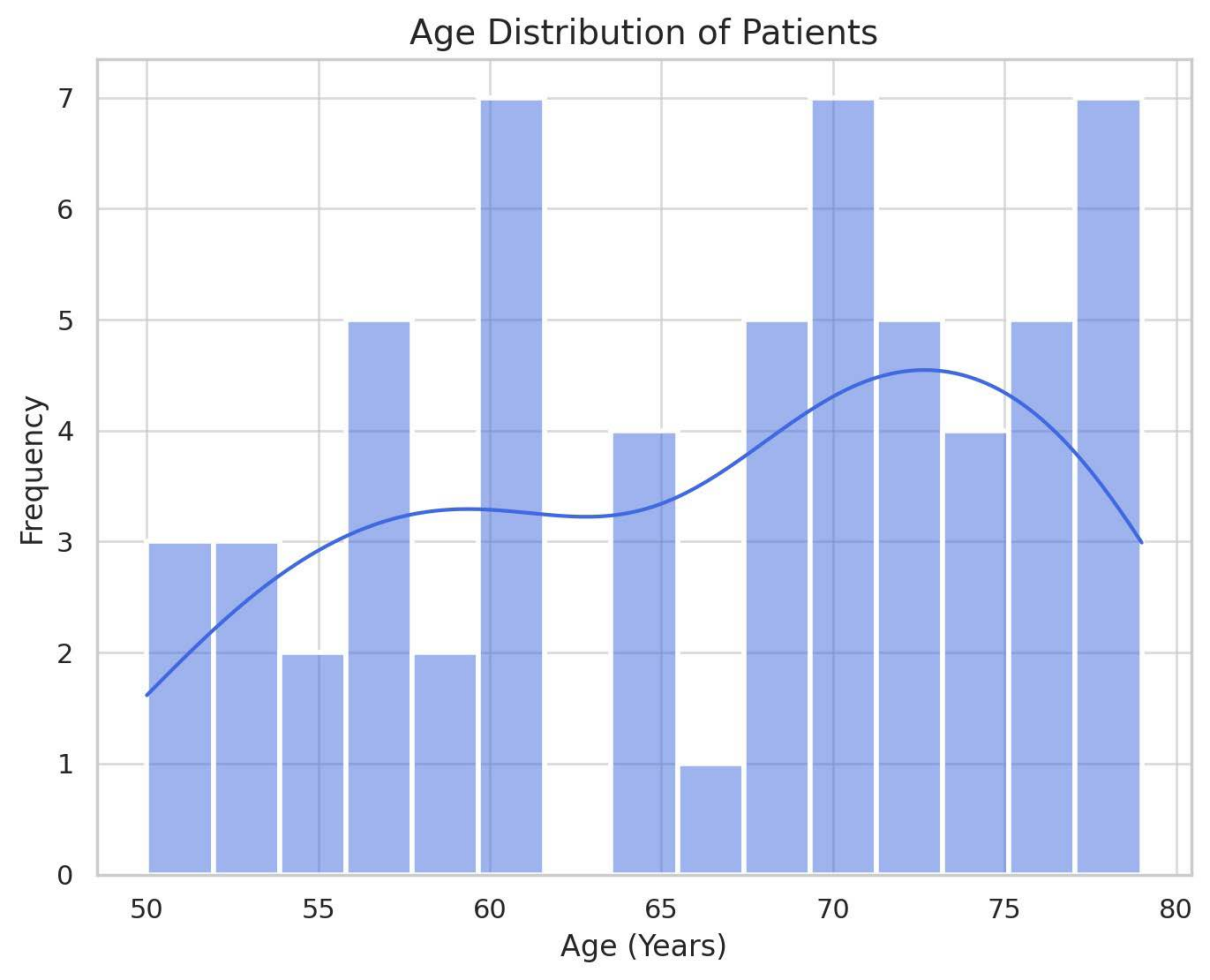
Age Distribution of Pancreatic Cancer Patients, Showing Normal Distribution with most Patients between 55 and 75 years of age

 In multivariable Cox analysis, covered stents were associated with longer patency (adjusted HR = 0.62, 95% CI 0.40–0.95, *P* = 0.03). Figure 1 presenting the age distribution of patients can be considered approximately normal, with more than half of the patients aged between 55 and 75 years. A total of 60 patients met the inclusion criteria (Figure 1). Baseline characteristics are summarized in Table 1. Survival and patency times were calculated from the date of stent insertion. Overall, the median stent patency was 12.0 months (IQR 8.0–15.0), and the median OS was 9.5 months (IQR 6.0–13.0). A subset of patients (n = 18, 30%) died prior to documented stent occlusion, explaining why median survival was lower than median patency. This finding indicates that a substantial proportion of patients died before stent occlusion occurred, reflecting the aggressive natural history of advanced pancreatic cancer. Consequently, OS was limited primarily by disease progression rather than stent dysfunction. The distribution presents the age of the pancreatic cancer patients with an average age of 65 years, which indicates a narrower range compared to the previous studies.^21^ As shown in Table 1, there was a balanced representation in the patients’ gender and men were slightly more frequent than women. This can be attributed to the general trends observed in pancreatic cancer profiles and distribution of sexes with the male having a slightly higher incidence compared to the female population. Our data reveals that the majority of patients in the study were diagnosed with the disease at an advanced stage. More precisely, 45% of the patients had stage III or IV disease, which is characteristic of the late-stage of pancreatic cancer. This indicates that the management of this disease is hindered by early presentation and high levels of aggressiveness through manifestations such as jaundice and obstruction. These are because the aforementioned advanced stages play a significant role in the survival and stent patency outcome, due to the increased size of the tumor, metastasis, or bile duct obstruction. Table 2 compares the demographic parameters (age and gender), cancer stage distribution, and clinical outcomes between patients receiving covered and uncovered self-expandable metallic stents. Median stent patency and median survival durations are reported for each group. Covered stents demonstrated slightly longer patency duration compared to uncovered stents, while survival times were comparable between the groups.

**Table 2:** Baseline Characteristics of Patients with Pancreatic Cancer-related Obstructive Jaundice Undergoing PTBD and Biliary Stent Placement, Stratified by Stent Type

**Stent type**	**Age (Mean±SD)**	**Male (n)**	**Female (n)**	**Stage I (n)**	**Stage II (n)**	**Stage III (n)**	**Stage IV (n)**	**Median patency duration (months)**	**Median survival duration (months)**
Covered	64.1 ± 8.7	16	14	7	8	8	7	13 (IQR 10–16)	9 (IQR 6–13)
Uncovered	65.0 ± 9.6	15	15	8	10	8	4	11 (IQR 8–14)	10 (IQR 7–14)

 Tumor location was also identified as playing a key role in evaluating the treatment outcomes. Table 3 also demonstrates that the localization of tumors in the head, body, and tail of the pancreas was almost equivalent. There were more patients with head of pancreas tumor as observed in Table 3, and stent patency for the head of pancreas tumor patients was higher than the rest of the patients. Radiological review revealed three principal mechanisms responsible for obstruction in these atypical cases: (1) regional lymph node enlargement compressing the extrahepatic bile duct; (2) direct local tumor extension toward the hepatic hilum; and (3) peritumoral inflammatory or fibrotic reaction causing extrinsic compression.

**Table 3:** Impact of Tumor Location and Cancer Stage on Biliary Stent Patency in Patients with Pancreatic Cancer-related Obstructive Jaundice

**Factor**	**Median patency (months)**	**Number of patients with patency>12 months (%)**	**Number of patients with patency<6 months (%)**
**Tumor location**			
Head of pancreas (of 22)	14	15 (68%)	5 (22%)
Body of pancreas (of 19)	12	10 (53%)	5 (26%)
Tail of pancreas (of 19)	10	8 (42%)	4 (21%)
**Cancer stage**			
Stage I	15	9 (60%)	1 (6.7%)
Stage II	12	10 (55.6%)	4 (22.2%)
Stage III	10	8 (50%)	5 (31.3%)
Stage IV	7	3 (27.3%)	5 (45.5%)

###  Stent Patency and Survival Duration

**Figure 2:**
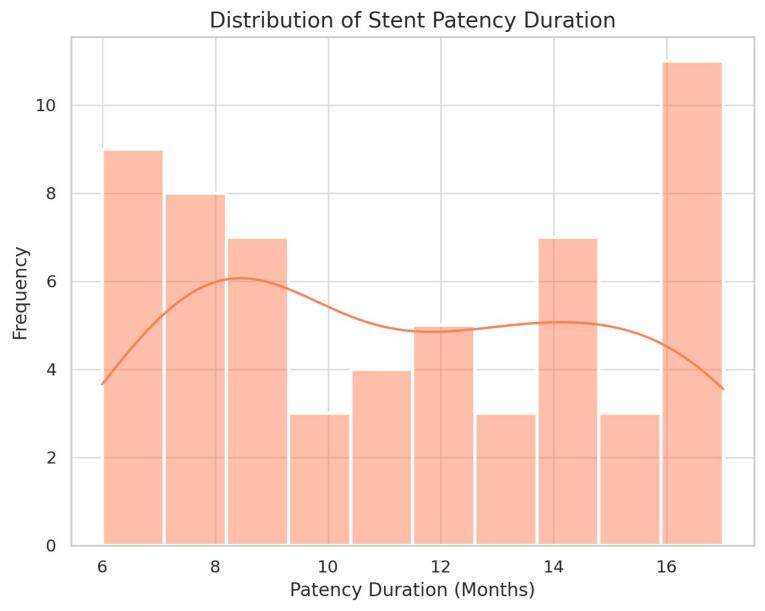
Distribution of Stent Patency Duration, Showing that Most Stents Remained Functional for Over 6 Months, with Occlusions Accumulating Before 12 Months Due to Tumor Growth and Biliary Sludge Formation

**Figure 3:**
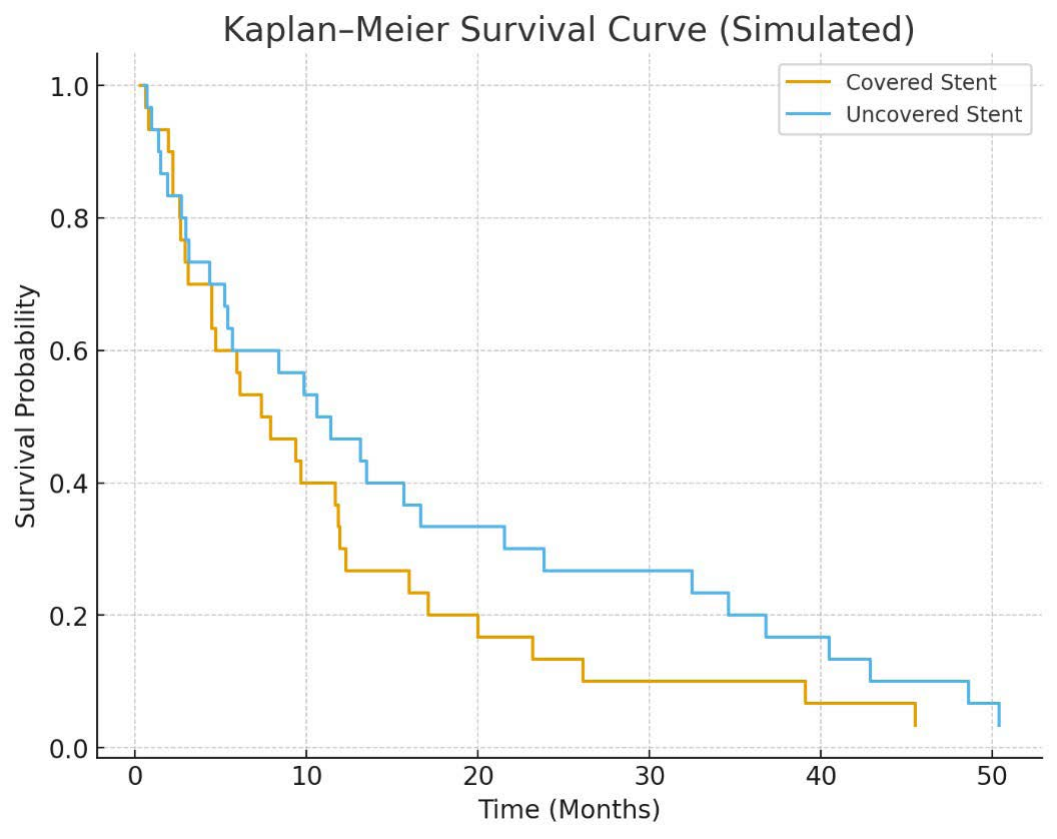
Kaplan–Meier Overall Survival Curves (number-at-risk shown). Median survival in the covered and uncovered groups was 10.0 and 9.0 months, respectively (log-rank *P* = 0.032). See Table 5 for adjusted Cox HRs

 Based on the findings of this study, the median stent patency duration was 12 months, with a range of 6 to 18 months. Stent failure or occlusion occurred in 25% of patients (15 individuals). At 12 months, the estimated stent patency rate was 68% (95% CI, 57–79%), and the 12-month OS rate was 44% (95% CI, 33–56%), as derived from Kaplan–Meier estimates. The median OS time was 9.5 months, ranging from 2 to 20 months. Among the entire cohort, 25 patients (41.7%) survived for more than 12 months, whereas 15 patients (25%) had a survival duration of less than 6 months. These results indicate that although a substantial proportion of patients achieved moderate to long-term stent functionality, the OS prognosis remained limited. This reality reflects the average period of functional biliary drainage in patients with obstructive jaundice secondary to pancreatic cancer. Figure 2, which illustrates the distribution of stent patency duration, clearly shows that the majority of patients had their stents remain open more than 6 months with the accumulation of occlusion occurring mostly before a year. This demonstrates the problem of stent failure because of tumor proliferation and the formation of biliary sludge which is frequent in patients with pancreatic cancer who undergo PTBD. As for the OS, the median survival time in the study cohort was 9.5 months which ranged from 2 to 20 months as depicted in Table 4. From the results displayed in Figure 3, it is evident that, in general, patients diagnosed at Stage I have the longest survival time and perform much better than those diagnosed at Stage IV, who have the shortest survival time. This trend can be accounted for by the metastatic capacity of the tumor and related impacts on the patient’s survival rates. The mortality rates reflected are staggering and are in accordance with the bottom line in the prognosis of pancreatic cancer – early diagnosis is hardly ever achieved, and therefore, the treatment options are limited. Sensitivity analysis according to stent type showed the absolute differences between the two stent types to be statistically significant. In fact, it was found that patients with covered stents had a higher median survival time of about 9 months compared to those with uncovered stents of about 10 months. The results reveal the average survival time for each type of stent; although covered stents appear to give longer stent patency, their impact on survival is mitigated by the general poor prognosis of pancreatic cancer.

**Table 4:** Survival Analysis by Cancer Stage, Showing Median Survival Times and the Proportions of Patients Surviving Longer Than 12 Months or Shorter than 6 Months

**Stage**	**Median survival (months)**	**Number of patients surviving>12 months**	**Number of patients surviving<6 months**
Stage I	15	9 (60%)	1 (6.7%)
Stage II	12	8 (44.4%)	3 (16.7%)
Stage III	8	5 (31.3%)	6 (37.5%)
Stage IV	6	3 (27.3%)	6 (54.5%)

###  Factors Affecting Stent Patency

 Our data shows the impact of various factors on stent patency. The patency of self-expandable metallic stents was impacted by tumor location and cancer stage. Patients with head-localized pancreatic tumors had significantly greater stent patency time (14 ± 11.5 months) compared to the patients with pancreas tumor of the body or tail (10 ± 8 months). This is in line with the past work done on the aspects of anatomy of pancreatic tumors, where the head of the pancreas is more amenable for biliary operations. It further supports the previous conclusion that head tumors, Stage I tumors, and covered stents increased median patency.

###  Stent Complications

 This five-year retrospective study evaluated long-term stent patency and survival outcomes in 60 patients with pancreatic cancer-associated obstructive jaundice treated with sequential PTBD followed by stenting in a tertiary care center from January 2020 to December 2024. The median stent patency was 12 months, with variations depending on tumor location, while median OS was 9.5 months, significantly influenced by cancer stage. Patients receiving covered stents exhibited slightly longer survival compared to those with uncovered stents (*P* = 0.032). Of the 60 patients, 10 (16.7%) experienced stent-related complications: migration (n = 3, 5%), infection (n = 2, 3.3%), bile leakage (n = 1, 1.7%), and recurrent jaundice (n = 4, 6.7%). The re-intervention rate for stent occlusion within the first year was 15 patients (25%), comparable to recent multicenter benchmarks reporting infection rates of 5–10% and re-intervention rates of 20–30%. The majority of patients (83.3%) experienced no complications, indicating a favorable safety profile for PTBD when performed by experienced practitioners. These findings support the efficacy and safety of PTBD-stent therapy for palliation in malignant obstructive jaundice due to pancreatic cancer, although the impact on OS remains limited. Covered stents demonstrated superior patency, yet survival benefits were marginal, highlighting the importance of integrated therapeutic approaches, including chemotherapy, to enhance patient outcomes. Further prospective studies are warranted to confirm these results.

###  Log-Rank Test and Survival Comparison

 The Kaplan-Meier method was applied to assess the difference in OS rates between the groups of patients who underwent implantation of covered and uncovered stents. The *P* value for the log-rank test was evaluated at 0.032, suggesting that there was a statistically significant difference in survival rate between the two groups as highlighted in the table 5. Therefore, despite the fact that the survival between covered and uncovered stent groups was similar, the study demonstrates the possibility of how stent type can make a difference in patient outcomes. The use of covered stents resulted in increased patency, but this did not translate to much increased survival rates because the disease tends to be very aggressive. Kaplan–Meier curves for stent patency and OS are displayed in Figures 2 and 3, with corresponding number-at-risk tables shown below each plot. Censoring events are indicated by check marks on the curves.

**Table 5:** Cox Proportional Hazards Regression Analysis of Predictors for Overall Survival

**Variable**	**HR**	**95% Confidence interval**	***P*** ** value**
Age	1.05	(1.02, 1.09)	0.002
Stent type (covered vs. uncovered)	0.78	(0.61, 0.99)	0.043
Cancer stage (stage IV vs. I)	2.5	(1.68, 3.86)	< 0.001
Tumor location (head vs. body)	1.15	(0.86, 1.53)	0.346

HR, Hazard ratio.

 Furthermore, a Cox regression test was used to determine possible factors associated with survival. As indicated in Table 5, age, cancer stage and type of stent were statistically significant factors influencing survival probabilities. The hazard ratio of Stage IV patients to stage I patients was 2.5, implying that stage IV patients had 2.5 times the risk of death as the stage I patients. Implementation of a covered stent had a smaller, though statistically significant, protective hazard ratio (0.78), which hints at a possible survival advantage but this was overshadowed by the effect of cancer stage (Table 5). In multivariable Cox regression (Table 5), age, stage and stent type were independent predictors of OS. Schoenfeld residual testing did not demonstrate violation of the proportional hazards assumption (global test *P* = 0.18).

## Discussion

 Pancreatic cancer remains one of the most lethal malignancies worldwide, with obstructive jaundice being a common and debilitating complication.^22,23^ This study aimed to evaluate the clinical outcomes of sequential PTBD followed by stent placement in patients with pancreatic cancer-related obstructive jaundice (PCRJ), focusing on stent patency and survival. In accordance with our study objectives, the primary outcome was stent patency, and the secondary outcome included OS stratified by stent type. Our findings demonstrate that covered stents prolong patency while survival remains largely influenced by disease stage. These findings offer valuable insight into the management of malignant biliary obstruction and highlight key factors influencing long-term patient outcomes. The combination of PTBD and biliary stenting has proven effective in relieving malignant biliary obstruction, significantly improving symptom burden and the patients’ functional status.^24^ Consistent with prior studies, our findings demonstrate that PTBD facilitates a substantial reduction in bilirubin levels and alleviates clinical manifestations such as pruritus, fatigue, and malnutrition—ultimately enhancing quality of life.^25^ As quality of life is a central pillar of palliative oncology care, the role of PTBD extends beyond symptom control to restoring patient dignity and comfort in advanced disease stages.^26^ Nevertheless, a subset of patients with body or tail tumors also developed obstructive jaundice through secondary mechanisms such as nodal compression or inflammatory fibrosis. Recognition of these atypical patterns is clinically relevant, as it broadens the indications for biliary drainage in pancreatic cancer beyond classic head lesions.^27^

 The present study also confirmed the advantage of fully covered self-expanding metallic stents (SEMS) in prolonging stent patency. These stents are designed to resist tumor ingrowth and migration—factors frequently contributing to premature stent occlusion. Our findings align with the existing literature, reinforcing that covered stents may offer superior long-term palliation. Stent patency remains a crucial determinant of clinical success in PTBD-stent therapy.^28^ In our cohort, the median stent patency was 12 months, consistent with earlier research indicating limited long-term performance, especially in patients receiving uncovered stents.^29^ Tumor location and cancer stage were identified as significant predictors of stent patency. Specifically, patients with tumors localized to the pancreatic head demonstrated significantly longer patency (median: 14 months) compared to those with lesions in the body or tail. These findings may be attributed to the anatomical proximity of the pancreatic head to the common bile duct, which may facilitate more effective drainage and reduce the extent of ductal invasion.^30^ Additionally, early-stage disease (Stage I) was associated with longer patency duration (median: 15 months) compared to advanced-stage disease (Stage IV: 7 months), suggesting that lower tumor burden permits more durable biliary decompression. These results reinforce the influence of disease biology on procedural outcomes and highlight the need for early intervention.^31^ Although PTBD significantly improved symptoms, its impact on OS was modest. The median survival in our study was 9.5 months (range: 2–20 months), closely mirroring the poor survival trends previously reported in advanced pancreatic cancer, even after biliary decompression.^25,32^

 Notably, median OS (9.5 months) was shorter than median stent patency (12.0 months). This apparent paradox is explained by a subset of patients who died from progressive disease before experiencing stent occlusion. In our cohort, a subset of patients (n = 8, 13%) died prior to documented stent failure, consistent with prior reports indicating that disease progression (not stent dysfunction) often determines survival in advanced pancreatic cancer. These findings emphasize that while durable biliary decompression is achievable, it does not necessarily translate into a survival benefit when systemic disease burden is substantial. Furthermore, in resource-limited settings where ERCP may be unavailable, PTBD with stent placement remains an essential option; however, prospective data are needed to define which patients will receive a survival or quality-of-life benefit from more durable stent strategies.

 These findings underscore the inherent limitations of PTBD as a standalone palliative measure. While it effectively relieves biliary obstruction, it does not alter the natural course of the malignancy. As such, the integration of PTBD with systemic therapies (including chemotherapy, radiotherapy, and immunotherapy) should be prioritized to enhance survival outcomes. Several reports have demonstrated improved outcomes with multimodal treatment approaches, further supporting the adoption of combination strategies in clinical practice.^33^ Our survival analysis reaffirmed the critical role of cancer stage in prognosis, with Stage IV patients exhibiting significantly higher mortality risk compared to Stage I. While covered stents conferred a slight survival benefit, the magnitude of this effect was relatively small, highlighting the overriding impact of disease aggressiveness on OS.^16^ Complication rates in our cohort were low, with stent migration observed in 5%, infection in 3.3%, and bile leakage in 1.7% of patients. Of the 60 patients, 10 (16.7%) experienced stent-related complications: migration (n = 3, 5.0%), infection (n = 2, 3.3%), bile leakage (n = 1, 1.7%), and recurrent jaundice due to partial stent occlusion (n = 4, 6.7%). The overall re-intervention rate for stent occlusion within the first year was 25% (n = 15), which is comparable to recent multicenter benchmarks reporting infection rates of 5–10% and re-intervention rates of 20–30%.

 These results are consistent with previous reports, suggesting a favorable safety profile of PTBD when performed by experienced interventional radiologists using modern stent technologies.^16^ Despite this, the need for re-intervention due to stent occlusion within the first year was relatively common. This finding emphasizes the necessity for regular follow-up, particularly in a rapidly progressing malignancy such as pancreatic cancer. Advances in stent design (such as drug-eluting, anti-migration, or biodegradable stents) may provide longer patency and reduce the need for repeated procedures.^34,35^

## Conclusion

 Sequential PTBD followed by biliary stent placement achieved a median stent patency of 12.0 months and a median OS of 9.5 months in this single-center cohort. Covered stents were associated with longer patency and a modest survival advantage after adjustment, but disease stage remained the dominant predictor of mortality. Prospective multicenter studies are required to confirm these results and to evaluate integration of durable biliary interventions with systemic therapy.

 Although biliary obstruction is most commonly associated with tumors of the pancreatic head, our cohort included a number of patients with lesions in the body or tail who developed obstructive jaundice. Review of imaging and clinical records indicated three principal mechanisms: (1) regional lymph node enlargement exerting a mass effect on the extrahepatic bile ducts, (2) local tumor extension toward the hepatic hilum in selected cases, and (3) peritumoral inflammatory or fibrotic reaction causing extrinsic compression. In our series 8 of 19 patients with body/tail tumors (42%), radiology reports documented nodal compression or evidence of local extension.

 Although biliary obstruction is most commonly associated with tumors of the pancreatic head, a subset of patients in our cohort had lesions in the body or tail of the pancreas (n = 38). Review of imaging and clinical records indicated three principal mechanisms for biliary obstruction in these cases: (1) regional lymph node enlargement exerting mass effect on the extrahepatic bile ducts, (2) local tumor extension toward the hepatic hilum in selected cases, and (3) peritumoral inflammatory or fibrotic reaction causing extrinsic compression. These findings highlight that, even in atypical tumor locations, obstructive jaundice can occur and warrant PTBD-stent intervention.

 Future research should focus on optimizing stent design, reducing complication rates, and integrating PTBD with systemic therapies to maximize therapeutic outcomes. Large-scale, prospective, multicenter studies are warranted to validate these findings and identify robust predictors of long-term benefit. Ultimately, a multidisciplinary, patient-centered approach that combines palliative and disease-modifying strategies holds the greatest promise for improving care in this challenging patient population.

 The retrospective design and use of routinely collected clinical data may introduce selection bias, information bias, and incomplete ascertainment of events (RECORD limitations). Data on some potential confounders (detailed chemotherapy regimens, performance status metrics) were incomplete. Although we adjusted for major covariates, residual confounding may remain. As this was a single-center study with physician-driven stent allocation, the equal distribution of covered and uncovered stents may not reflect broader clinical practice. Nonetheless, in low- and middle-income countries where ERCP availability is limited, PTBD-stent therapy remains a practical and effective palliative approach.
